# Supplementary material for: Multiplex Digital PCR to Detect Amplifications of Specific Androgen Receptor Loci in Cell-Free DNA for Prognosis of Metastatic Castration-Resistant Prostate Cancer
Source: Cancers (Basel). 2020 Aug 1;12(8):2139. doi: 10.3390/cancers12082139 (PMC7465398; doi:10.3390/cancers12082139)
Supplement: Supplementary file 1 [file cancers-12-02139-s001.pdf]

Supplementary Materials:

Multiplex Digital PCR to Detect Amplifications of Specific Androgen Receptor Loci in Cell-Free DNA for Prognosis of Metastatic Castration-Resistant Prostate Cancer

Meijun Du, Chiang-Ching Huang, Winston Tan, Manish Kohli and Liang Wang

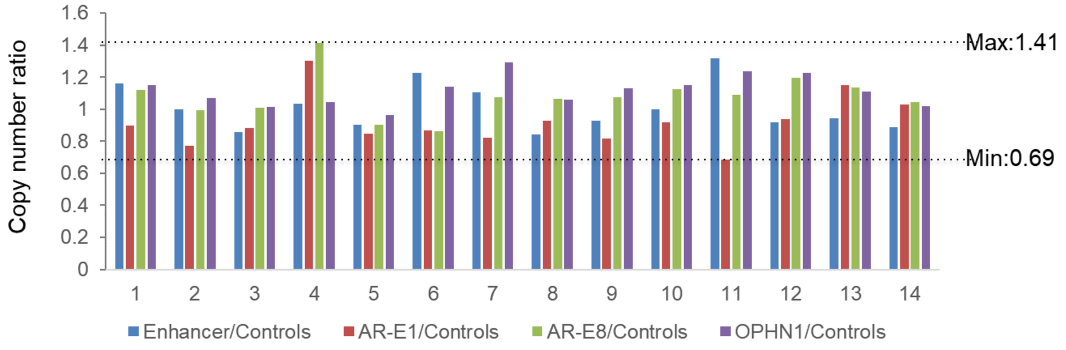

Figure S1. Distribution of CN ratio of *AR*-En, *AR*-E1, *AR*-E8, and *OPHN1* in 14 gDNA controls. Mean CN ratio for each locus is 1.01 (Range 0.84–1.32) for *AR*-En, 0.92 (Range 0.69–1.30) for *AR*-E1, 1.08 (Range 0.86–1.41) for *AR*-E8, and 1.12 (Range 0.96–1.29) for *OPHN1.*

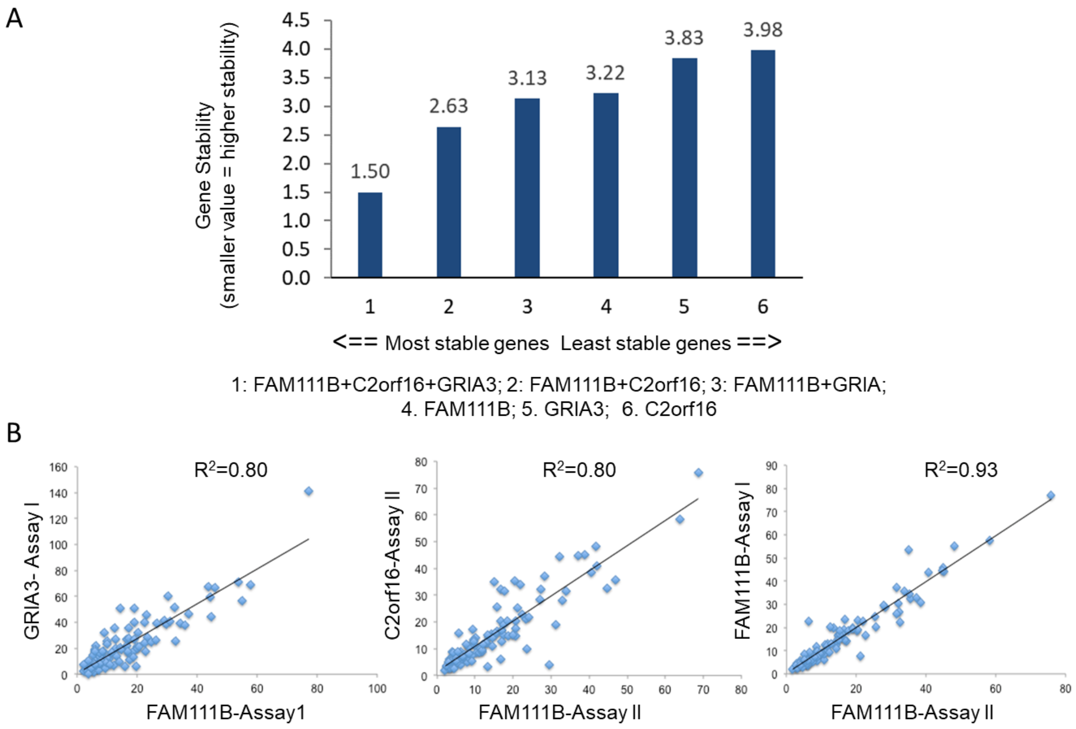

Figure S2. Stability of reference control genes (**A**) and correlation of different controls in Assay I and Assay II (**B**).

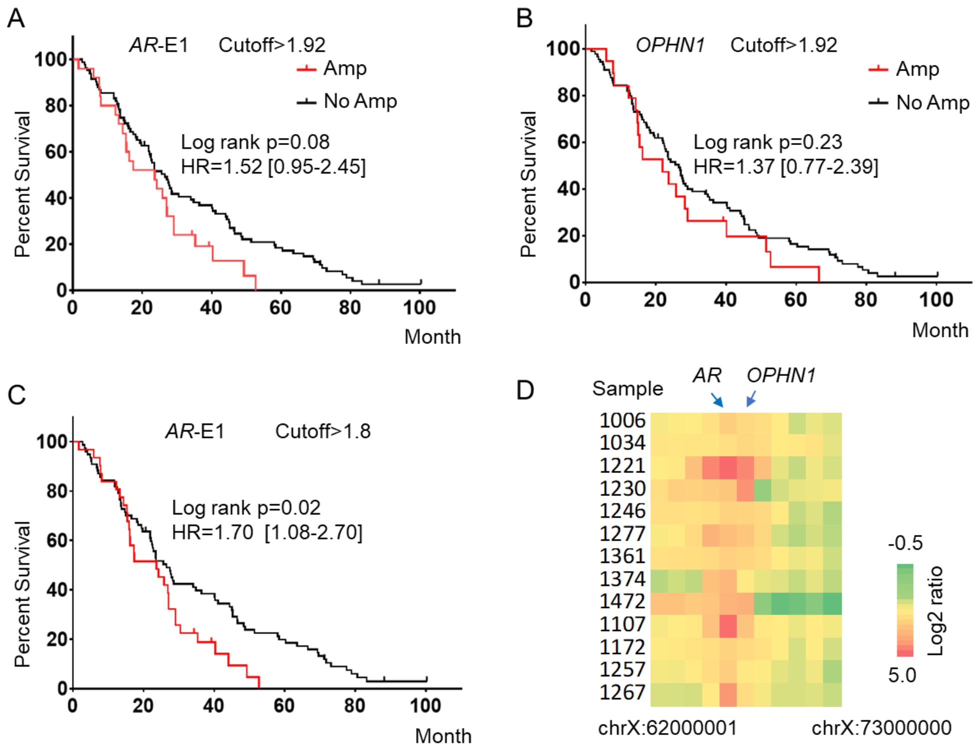

Figure S3. Heatmap of the amplification of *AR* loci (1Mb bin) and association of amplification of *AR*-E1 and *OPHN1* with OS. (**A**). Heatmap showed the amplification of *AR* (1Mb bin covering *AR*-En, *AR*-E1, and *AR*-E8) in 13 samples with co-amplification of *AR*-En, *AR*-E1, and *AR*-E8. Association of amplification of *AR*-E1 and *OPHN1* with OS. Although showing clear trend of poor OS, the amplifications at the two sites (**B**,**C**) do not reach statistical significance when cutoff > 1.92. (**D**). *AR*-E1 showed significant association with OS when the cutoff of amplification call > 1.8.

Table S1. Sequences of the primers and TaqMan probes used for dPCR.

Primers or Probes	Sequences 5'-3'	Length (bp)	PCR Product (bp)
C2orf16 For	5'-CAGCTGAACTGATGGAACCT-3'	20	
C2orf16 Probe	5'-CCTGCCAGAGAAGGGTCCAGTTAC-3'	24	
C2orf16 Rev	5'-GACCACAGAAGGCTGAGAAATA-3'	22	74
FAM111B For	5' -AGCAAATGTGCGAAGGTAAC- 3'	20	
FAM111B Probe	5'-ACAGAGTTCTGCCCTACTCCTGACA-3'	25	
FAM111B Rev	5'-CATGGCTCAATGGAAAACC-3'	19	77
Enhancer For	5'-CAGAACCCACCTGCTACTAC-3'	20	
Enhancer Probe	5'-ACTATGGCCCTCCATTCATGCAACT-3'	25	
Enhancer Rev	5'-CTCTGTGCCATTCACTCCAT-3'	20	67
AR Exon1 For	5'-CCTCCAAGGACAATTACTTAGGG-3'	23	
AR Exon1 Probe	5'-CGACCATTTCTGACAACGCCAAGG-3'	24	
AR Exon1 Rev	5'-GACACCGACACTGCCTTAC-3'	19	79
AR Exon8 For	5'-AGTGCCCAAGATCCTTTCTG-3'	20	
AR Exon8 Probe	5'-AGTCAAGCCCATCTATTTCCACACCC-3'	26	
AR Exon8 Rev	5'-TAGGGTTTCCAATGCTTCACT-3'	21	71
OPHN1 For	5'-GAATGGAGTCTTGTGATCTAGGG-3'	23	
OPHN1 Probe	5'-CCTCTCTGCCCTGGAGTTTGTCAC-3'	24	
OPHN1 Rev	5'-TGTAAGCCTATGCTTGTTCAGT-3'	22	71

Abbreviations: For: forward; Rev: reverse.

© 2020 by the authors. Licensee MDPI, Basel, Switzerland. This article is an open access article distributed under the terms and conditions of the Creative Commons Attribution (CC BY) license (http://creativecommons.org/licenses/by/4.0/).
